# Scalp acupuncture alleviates cerebral ischemic stroke-induced motor dysfunction in rats via regulating endoplasmic reticulum stress and ER-phagy

**DOI:** 10.1038/s41598-023-36147-8

**Published:** 2023-06-21

**Authors:** Yuxin Zhang, Huijuan Lou, Jing Lu, Xiaolei Tang, Tingting Pang, Siyuan Lei, Deyu Cong, Yufeng Wang, Liwei Sun

**Affiliations:** 1 Research Center of Traditional Chinese Medicine, The Affiliated Hospital to Changchun University of Chinese Medicine, Changchun, Jilin People’s Republic of China; 2 Key Laboratory of Active Substances and Biological Mechanisms of Ginseng Efficacy, Ministry of Education, Changchun, Jilin People’s Republic of China; 3 Massage Department, The Affiliated Hospital to Changchun University of Chinese Medicine, Changchun, Jilin People’s Republic of China; 4 Changchun University of Chinese Medicine, Changchun, Jilin People’s Republic of China

**Keywords:** Cell death in the nervous system, Stroke

## Abstract

Cerebral ischemic stroke is a high-risk disease and imposes heavy burdens on patients in china. Acupuncture has been used for thousands of years to treat motor dysfunction, cognitive disorder and language barrier caused by cerebral ischemic stroke. Acupoint lines, vertex middle line and anterior oblique line of vertex temple, are always employed to treat cerebral ischemic stroke. However, the mechanism of the two acupoint lines in relieving cerebral ischemic stroke needs further exploration. In the present study, scalp acupuncture treatment alleviated the motor dysfunction, brain damage, and cell death induced by middle cerebral artery occlusion (MCAO) in rats. Proteomics analysis and ultrastructure observation indicated that endoplasmic reticulum and lysosomes might involve in the mechanism of the scalp acupuncture treatment in suppressing MCAO-triggered neural deficits. Effect of the scalp acupuncture treatment on ER stress was then investigated and found that the activation of ER stress mediators, including PERK, IRE1, and ATF6, was downregulated after the scalp acupuncture treatment. Co-localisation analysis of KDEL and CD63 showed that the engulfment of ER fragments by lysosomes was accelerated by the scalp acupuncture treatment. Moreover, expression of pro-apoptotic protein CHOP, phosphorylated-JNK, cleaved capases-3 and -9 also decreased after the scalp acupuncture. In conclusion, the present study showed that scalp acupuncture of vertex middle line and anterior oblique line of vertex temple may alleviate cerebral ischemic stroke by inhibiting ER stress-accelerated apoptosis.

## Introduction

In China, thousands of people die from cerebral ischemic stroke every year, while those who survive suffer from hemiplegia, spasm, aphasia, and cognitive disorders^1^. Ischemic stroke is caused by reduced blood flow that prevents adequate supplies of nutrients and oxygen from reaching brain tissues, resulting in brain neuronal injury that ultimately leads to cell apoptosis and nervous system dysfunction^2,3^. Nowadays, researchers are focusing on post-ischemic cell apoptosis as a potential target of therapies under development for alleviation of neurological deficits caused by ischemic stroke^2^.

In healthy cells, the endoplasmic reticulum (ER) is responsible for synthesis of proteins, lipids, and carbohydrates^4^. However, cerebral ischemic stroke-induced accumulation of misfolded proteins has been reported to trigger excessive ER stress pathway activation^5,6^. In turn, ER stress pathway activation triggers the unfolded protein response (UPR) pathway that either restores ER homeostasis or leads to cell death via apoptosis^5,7^. The UPR pathway includes three key proteins, inositol-requiring protein-1 (IRE1), protein kinase R-like endoplasmic reticulum kinase (PERK), and activating transcription factor-6 (ATF6)^8^. IRE1 evokes neuronal apoptosis by activating c-Jun NH2-terminal kinase (JNK), while PERK and ATF6 may also trigger cell apoptosis via the C/EBP homologous protein (CHOP) pathway^9,10^.

Importantly, ER stress activates the autophagy pathway, a pathway that degrades and removes damaged ER components via a process known as ER-phagy^11^. Mechanistically, ER stress induces ER-phagy receptor activation that results in ER-phagy receptor binding to autophagy marker microtubule-associated protein light chain 3B (LC3B)^11^. In recent years, several ER-phagy receptors have been identified, such as family with sequence similarity 134, member B (FAM134B), reticulon 3 long (RTN3L), cell-cycle progression gene 1 (CCPG1), SEC62 homologue (SEC62), testis-expressed 264 (TEX264), and atlastin 3 (ATL3)^12^. FAM134B, the most extensively studied receptor, associates with LC3B/GABARAPL2 on the autophagosomal membrane to thereby trigger autophagosomal engulfment of ER debris^13^. Thereafter, autophagosomes transfer ER debris to lysosomes, resulting in formation of autolysosomes that complete the ER-degradative process^14^.

In China, acupuncture has been used to treat nervous system dysfunction-related diseases for thousands of years, due to its reported abilities for alleviating stroke-induced disability, neuropathic pain, vascular dementia, and so on^15–18^. Traditionally, acupuncture involves insertion of thin metal needles into suitable acupoints or acupoint lines to relieve specific maladies^19^. In this study, acupuncture needles were inserted at sites located along the scalp middle line of the vertex and the anterior parietotemporal oblique line in order to alleviate motor dysfunction associated with middle cerebral artery occlusion (MCAO)-induced brain damage in rats. The two acupuncture lines are viewed as an established clinical practice for improving hemiplegia, paralysis, and spasm caused by cerebral infarction^20–22^. However, potential mechanism(s) underlying observed neuroprotective effects of scalp acupuncture therapy are unknown, prompting this investigation.

## Results

### Scalp acupuncture improved neurological and motor functions of rats with MCAO-induced brain damage

**Figure 1:**
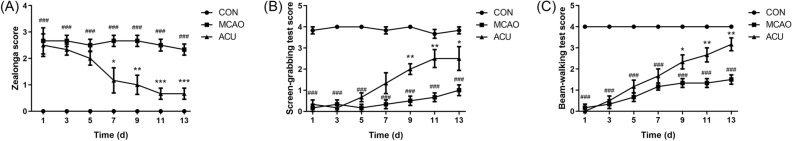
Scalp acupuncture improved neurological function of rats with MCAO-induced brain damage. Several neurological deficit scales were used to interpret results. (**A**) Zea Longa score. (**B**) Screen-grabbing test score. (**C**) Beam-walking test score. Results represent triplicate independent experiments and are expressed as the mean ± standard deviation (n = 6). ^###^p < 0.001 (MCAO vs CON); *p < 0.05, **p < 0.01, ***p < 0.001 (ACU vs MCAO).

Measurements of neurological and motor deficits were conducted and interpreted based on several neurological deficit reference scales in order to judge the effect of acupuncture treatment on ischemic stroke-induced neurological dysfunction. According to the results (Fig. 1), after scalp acupuncture treatment of rats with MCAO-induced brain damage for 7–9 days, markedly improved neurological function was observed as compared to that of untreated MCAO group rats.

### Scalp acupuncture alleviated tissue damage in brains of rats with MCAO-induced brain damage

**Figure 2:**
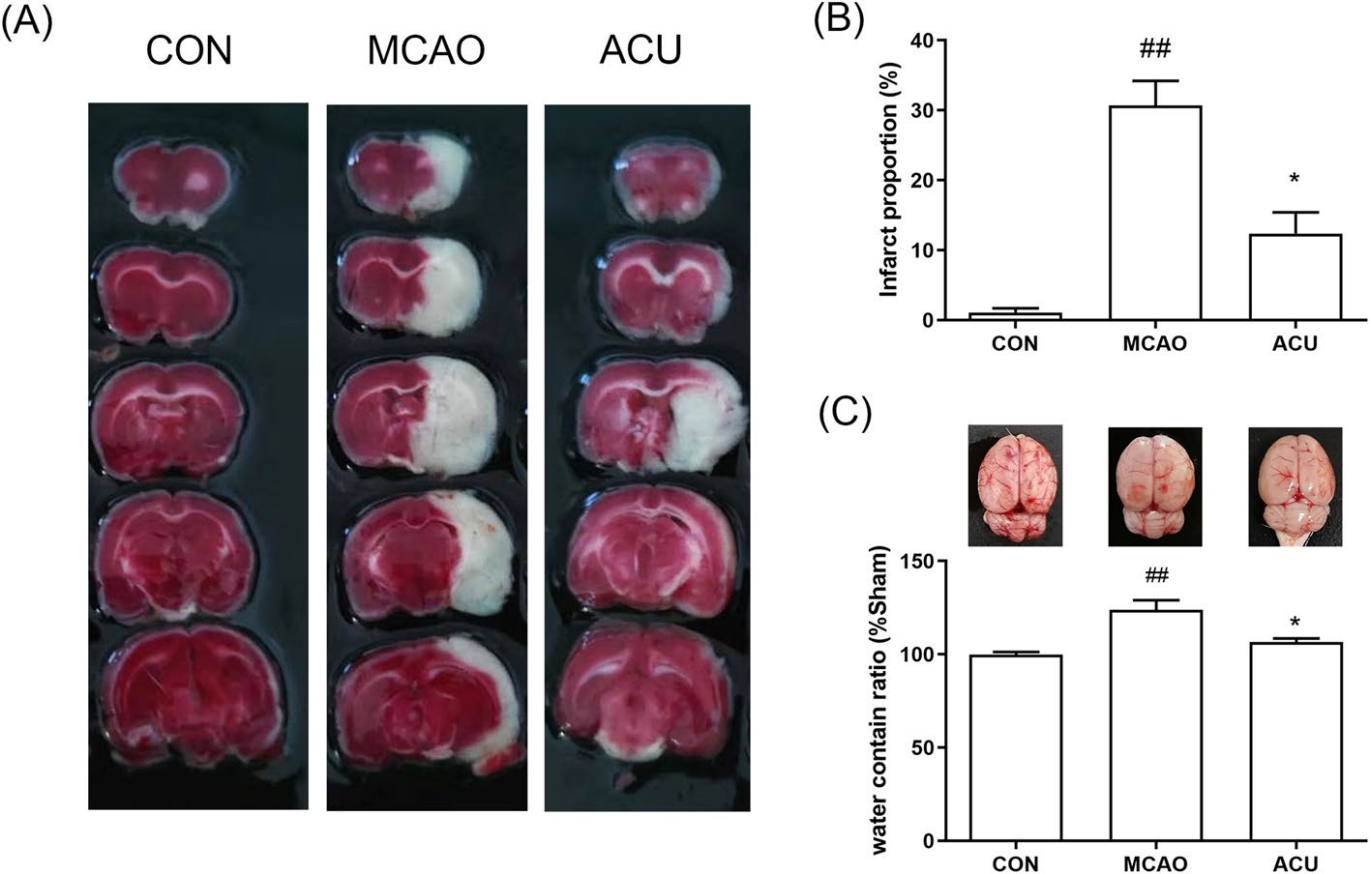
Scalp acupuncture alleviated tissue damage in brains of rats with MCAO-induced brain damage. (**A**) TTC staining results. (**B**) Infarct areas, as calculated using ImageJ. (**C**) Brain water content was measured to evaluate the extent of edema. Results represent triplicate independent experiments and are expressed as the mean ± standard deviation (n = 4–5). ^##^p < 0.01 (MCAO vs CON); *p < 0.05 (ACU vs MCAO).

**Figure 3:**
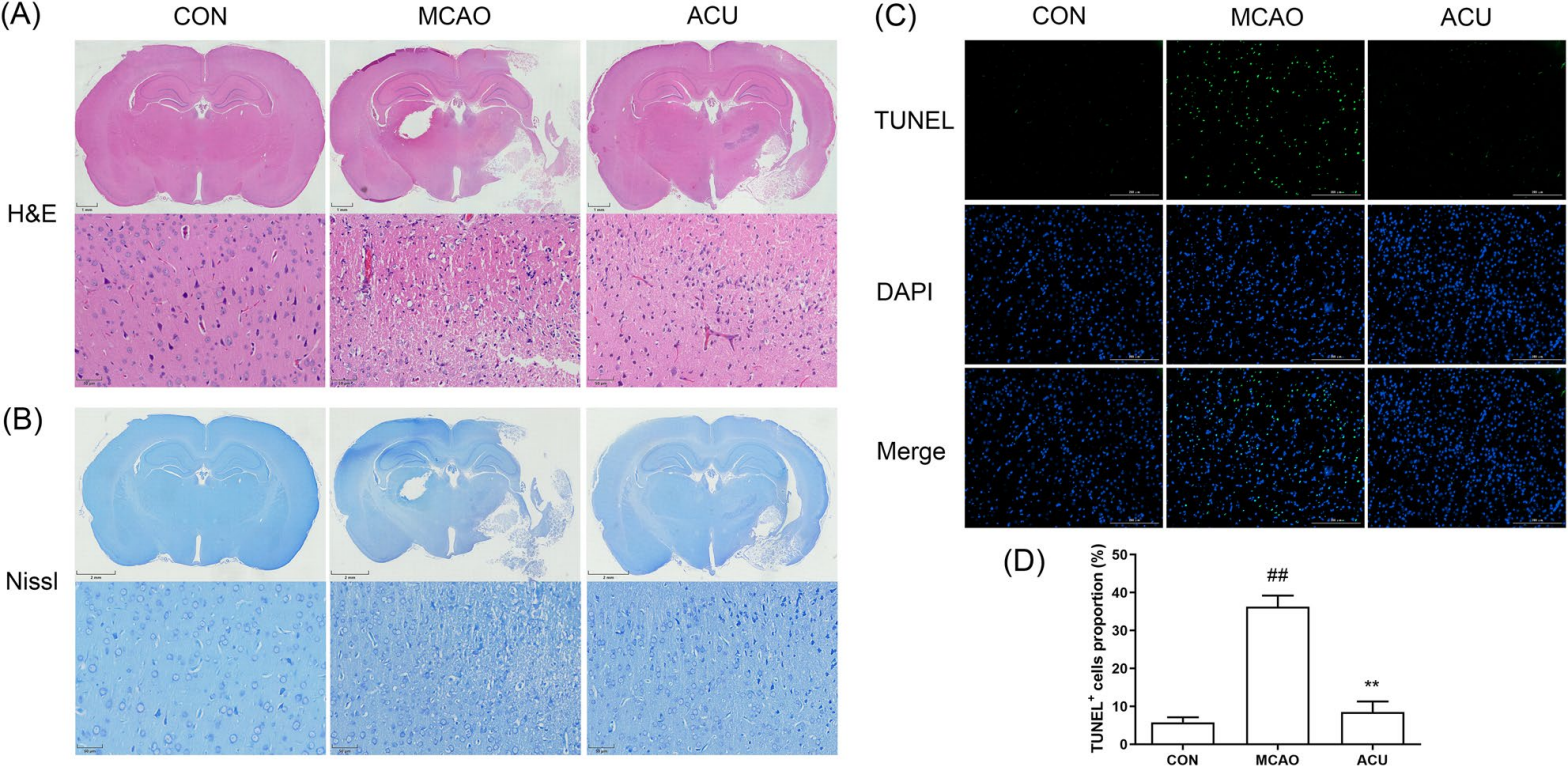
Scalp acupuncture reduced MCAO-induced neuronal apoptosis. (**A**) H&E staining and (**B**) Nissl staining results obtained for brain tissue sections based on photographs of specimens as viewed using an optical microscope. (**C**) Apoptotic cells in brains as detected using TUNEL staining. (**D**) Numbers of apoptotic cells, as calculated using ImageJ. Results represent triplicate independent experiments and are expressed as the mean ± standard deviation (n = 4). ^##^p < 0.01 (MCAO vs CON); **p < 0.01 (ACU vs MCAO).

Ischemia usually leads to brain infarction and edema. In Fig. 2A,B, 2,3,5-triphenyltetrazolium chloride (TTC) staining results indicated that the MCAO-induced white infarct area was reduced by acupuncture treatment. As shown in Fig. 2C, MCAO-induced brain edema was also reduced after acupuncture treatment.

### Scalp acupuncture reduced neuronal MCAO-induced injury

To determine whether scalp acupuncture treatment alleviated MCAO-induced neural damage, H&E staining, Nissl staining, and terminal deoxynucleotidyl transferase biotin-dUTP nick end labeling (TUNEL) staining assays were performed. Results of H&E staining and Nissl staining assays (Fig. 3A,B) revealed that cortex of MCAO group rat was severely damaged, as evidenced by apparent cellular shrinkage and disordered cellular arrangement within the penumbra region. However, scalp acupuncture treatment mitigated MCAO-induced neural damage. Similarly, TUNEL staining assay results (Fig. 3C,D) revealed that acupuncture treatment decreased MCAO-triggered cell apoptosis within the penumbra region of cortex. Collectively, these results demonstrated that scalp acupuncture provided a neuroprotective effect.

### Proteomics analysis of brain proteins of acupuncture-treated and untreated rats with MCAO-induced brain damage

**Figure 4:**
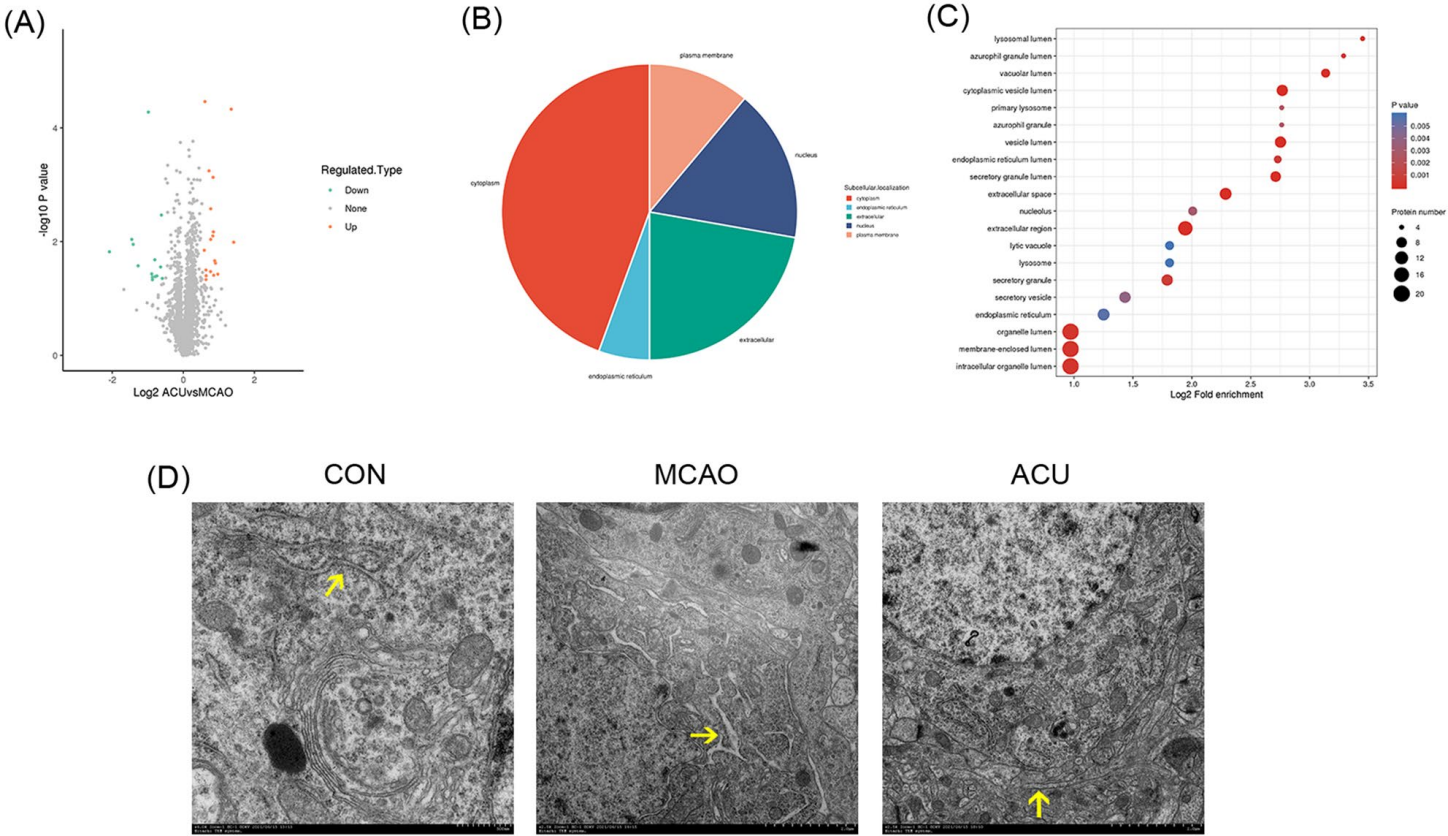
Proteomics analysis of brain proteins of MCAO group and ACU group rats. Brain protein differences between MCAO and ACU groups of rats were compared, as shown in (**A**) volcano plot, (**B**) subcellular localization, and (**C**) cellular component. (**D**) ER ultrastructure (indicated by arrow) as assessed via TEM (6000 × magnification). Results represent triplicate independent experiments.

To investigate proteomic differences between brains of MCAO group and ACU group rats, proteomics analysis of brain samples was performed. A total of 32 different brain proteins were detected between MCAO and ACU groups, including 18 up-regulated proteins and 14 down-regulated proteins (Fig. 4A). Results obtained regarding protein subcellular localisation and cellular component (Fig. 4B,C) suggested that scalp acupuncture treatment modulated ER-associated processes, as supported by functional terms obtained for the 14 proteins related to ER lumen, ER component, lysosome, and endocytosis. Subsequent ultrastructural observations of ER organelles of each group revealed that MCAO induction led to ER swelling that was reversed by acupuncture treatment (Fig. 4D). It is well known that UPR pathway activation leads to increased ER size that can be reversed by triggering of ER-phagy^23^. Based on results of previously published recent studies and results presented here, we speculate that the mechanism underlying observed therapeutic effects of scalp acupuncture treatment likely involve ER stress-related pathway activities.

### Scalp acupuncture mitigated MCAO-induced ER stress

**Figure 5:**
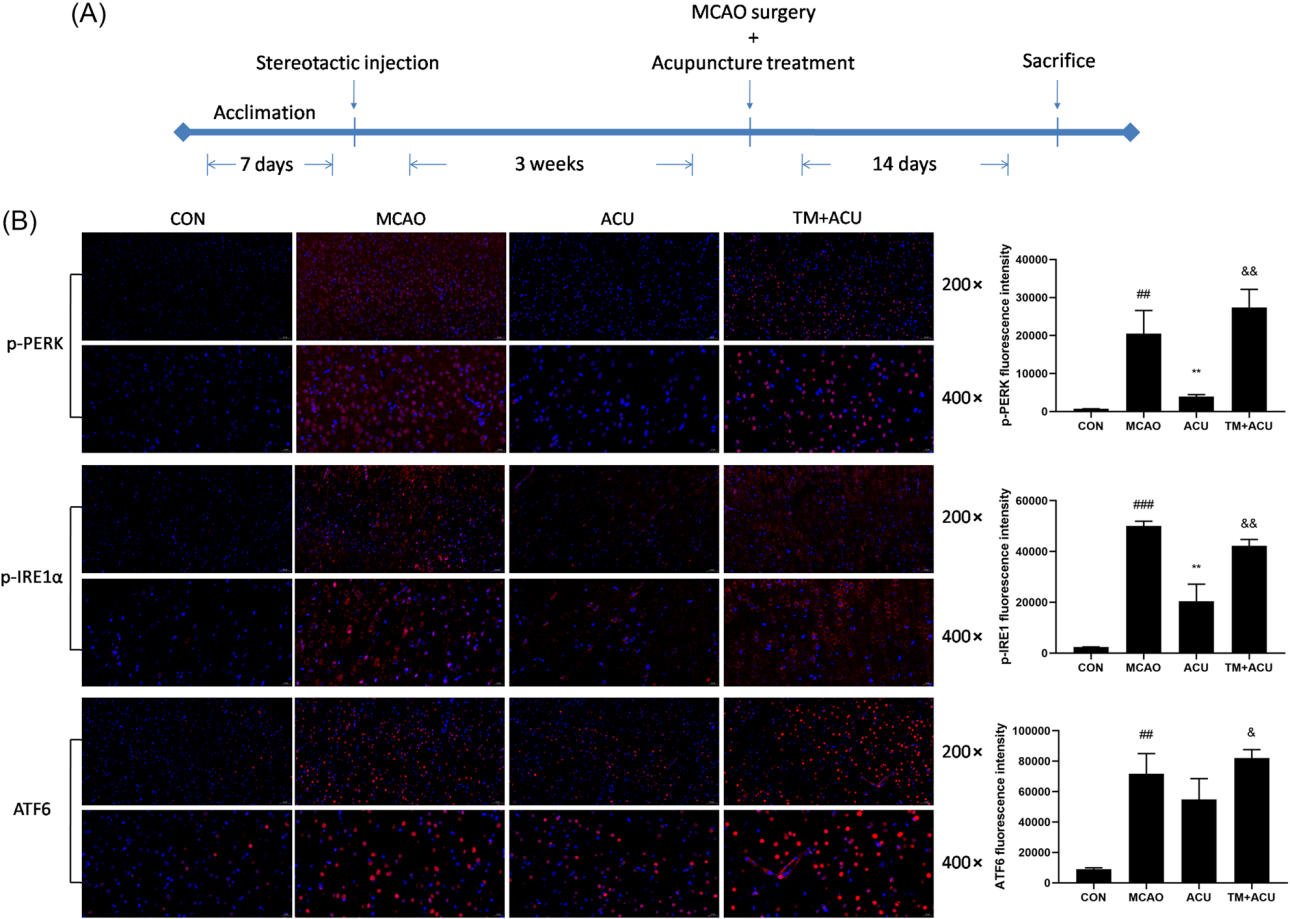
Scalp acupuncture mitigated MCAO-induced ER stress. (**A**) Design of the experiment. (**B**) Immunofluorescence (red) of p-IRE1, p-PERK, and ATF6 proteins shown at 200 × magnification (scale = 50 μm) and 400 × magnification (scale = 20 μm). Results represent triplicate independent experiments and are expressed as the mean ± standard deviation. ^##^p < 0.01, ^###^p < 0.001 (MCAO vs CON); **p < 0.01 (ACU vs MCAO); ^&^p < 0.05, ^&&^p < 0.01 (TM + ACU vs ACU).

To test our conjecture, triggering of ER stress (based on detection of p-IRE1, p-PERK, and ATF6) was assessed in brain tissues via immunofluorescence. TM, an activator of ER stress, was injected into brain cortex sites of TM + ACU group rats before MCAO surgery (Fig. 5A). As shown in Fig. 5B, the fluorescence intensity of cells expressing p-IRE1, p-PERK, and ATF6 proteins increased after MCAO induction then decreased after subsequent acupuncture treatment. However, the therapeutic effect of acupuncture treatment was abolished by TM.

### Scalp acupuncture enhanced ER-phagy

**Figure 6:**
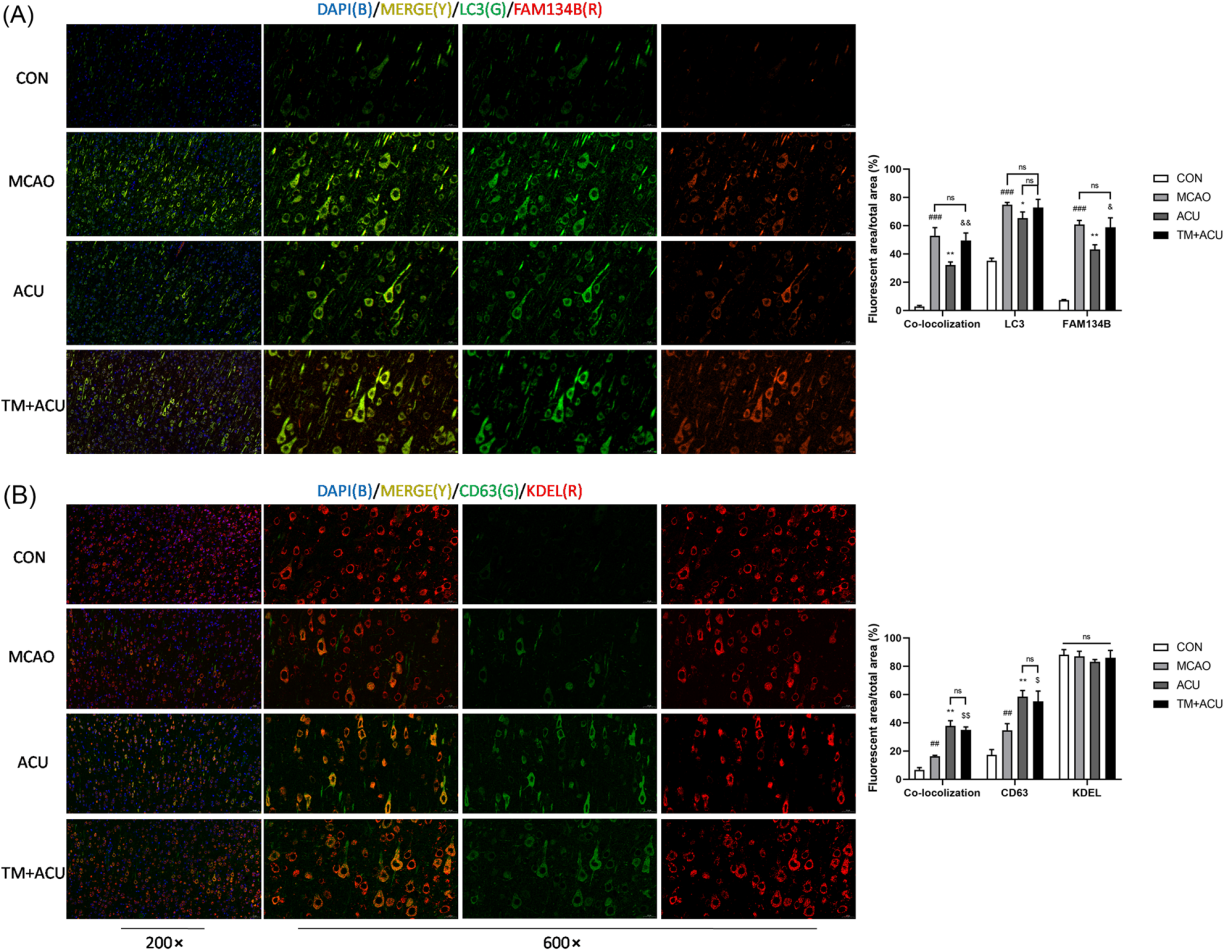
Scalp acupuncture enhanced ER-phagy. (**A**) Co-localisation of LC3 with FAM134B. (**B**) Co-localisation of CD63 with KDEL. Pictures of cortex were obtained at 200 × magnification (scale = 50 μm) and 600 × magnification (scale = 20 μm). ^##^p < 0.01, ^###^p < 0.001 (MCAO vs CON); *p < 0.05, **p < 0.01 (ACU vs MCAO); ^&^p < 0.05, ^&&^p < 0.01 (TM + ACU vs ACU); ^$^p < 0.05, **,^$$^ < 0.01 (TM + ACU vs MCAO); ns is short for no significance.

FAM134B is a transmembrane ER receptor protein that maintains ER homeostasis by interacting with LC3, a mediator of autophagy^13^. Immunofluorescence results revealed increased expression levels of FAM134B and LC3 proteins in brain cells of MCAO group rats that were reduced after subsequent acupuncture treatment (Fig. 6A), while TM increased the expression of FAM134B and LC3. In order to investigate whether scalp acupuncture facilitates lysosome to engulf ER fragments, we observed the co-localisation of CD63 (a marker of lysosome) and KDEL (a marker of ER). Intriguingly, the results shown in Fig. 6B indicate that acupuncture treatment led to enhanced lysosome formation and co-localisation of CD63 and KDEL, whereas TM didn’t affect acupuncture’s effect.

### Scalp acupuncture diminished ER stress-induced apoptosis

**Figure 7:**
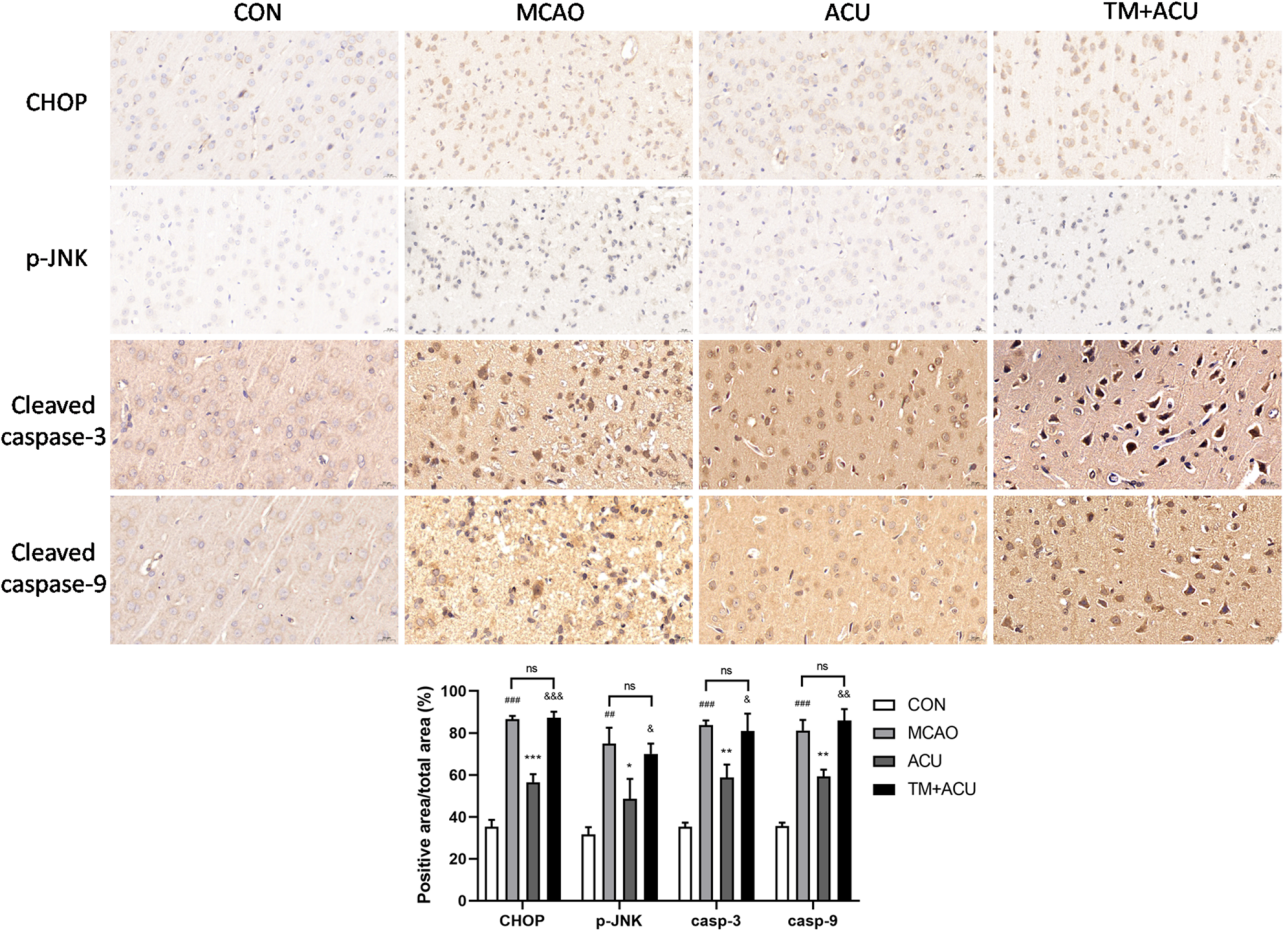
Scalp acupuncture treatment reduced ER stress-induced apoptosis. Immunohistochemical detection of intracellular proteins CHOP, p-JNK, cleaved caspase-3, and cleaved caspase-9, with images of cortex obtained at 400 × magnification (scale = 20 μm). ^##^p < 0.01, ^###^p < 0.001 (MCAO vs CON); *p < 0.05, **p < 0.01, ***p < 0.001 (ACU vs MCAO); ^&^p < 0.05, ^&&^p < 0.01, ^&&&^p < 0.001 (TM + ACU vs ACU); ns is short for no significance.

Levels of key mediators of apoptosis, cleaved caspase-3 and cleaved caspase-9, were determined via immunohistochemical analysis. As shown in Fig. 7, acupuncture treatment suppressed MCAO-induced cellular expression of cleaved caspase-3 and cleaved caspase-9. Due to the fact that CHOP and JNK have been reported to participate in ER stress-triggered cell apoptosis^7^, intracellular CHOP and p-JNK levels were also assessed. As shown in Fig. 7, acupuncture treatment inhibited MCAO-induced CHOP and p-JNK expression. However, cortical TM injection nullified scalp acupuncture-induced suppression of apoptosis.

## Discussion

Scalp acupuncture has been clinically administered as a therapeutic treatment for cerebral ischemic stroke for many years in China^24,25^. Importantly, traditional Chinese medicine (TCM) dictates that stroke-induced symptoms are caused by brain damage^24^. Based on this premise, TCM practitioners have evaluated numerous scalp acupoints to assess their functions, resulting in classification of scalp areas and acupuncture point lines into motor, visual, language, and other categories based on therapeutic effect^26^. Based on the clinical experience of our team, we administered acupuncture treatment to rats with MCAO-induced brain damage using the vertex middle line and the anterior oblique line of the vertex temple in order to alleviate the type of motor dysfunction induced by our rat stroke model (stroke-induced paralysis or limb spasm). In this study, scalp acupuncture treatment reduced neural deficits and brain damage induced by MCAO. Based on results of TUNEL staining and proteomics results obtained here, we conjectured that MCAO-induced brain damage was likely related to ER stress-induced neuronal cell death.

It has been reported that ER stress is likely initiated by cerebral ischemic stroke^27,28^. In cells in a resting state, ER stress UPR pathway effector proteins (e.g., PERK, IRE1, ATF6) are bound to GRP78/BIP, which restricts their activities until they are released from binding to GRP78/BIP, as occurs when cells are stimulated by effects of ischemic stroke^5,29^. Once released, PERK undergoes autophosphorylation then phosphorylates eukaryotic translation initiation factor 2α (eIF2α), which then activates pro-apoptotic protein CHOP^5^. Meanwhile, ischemic stroke also triggers ATF6 expression followed by ATF6 translocation into the Golgi apparatus. Once ATF6 enters the Golgi apparatus, it is cleaved and thereby activated^9,28^. Thereafter, activated ATF6 enters the nucleus, where it up-regulates CHOP expression^30^. Concurrently, IRE1 is phosphorylated, which triggers apoptosis via the IRE1/ASK1/JNK cascade^8,31^. Intriguingly, neuronal apoptosis has been reported to play a critical role in ischemic stroke-induced motor dysfunction, with ER stress induced by TM administration shown to induce more severe sensory and motor deficits^27^. Here, scalp acupuncture treatment reduced ischemic stroke-evoked ER stress and cell apoptosis by regulating CHOP and JNK signaling, while TM administration abolished these acupuncture therapeutic effects. Notably, these results collectively suggest that scalp acupuncture treatment elicited neuroprotection by inhibiting ER stress, as consistent with some results obtained in an excellent previously reported study utilizing acupuncture to treat ischemic stroke rats^27^. However, in that work they did not investigate whether apoptosis or ER-phagy was involved in the observed neuroprotective effect, prompting us to conduct additional experiments here to answer this question.

When under stress, the ER invokes two different pathways to maintain ER function and cellular homeostasis: ER-associated protein degradation (ERAD) and ER-phagy^8,11,12^. ERAD is a process by which the ER allows misfolded proteins to accumulate until they trigger the ubiquitin–proteasome system response, resulting in removal of the dysfunctional proteins from the cell^8^. In contrast, ER-phagy, a process evoked by specific receptors, leads to up-regulated expression of some of these receptors to accelerate removal of abnormal ER components via LC3-mediated autophagy (macro-ER-phagy) to support cellular recovery^11^. A key ER-phagy receptor, FAM134B, has been extensively studied and was the first such receptor to be identified^32^. Notably, knockdown of FAM134B expression has been shown to lead to ER expansion, defective ER degradation, and reduced macro-ER-phagy pathway activation^11,13^. Recently, more and more ER-phagy receptors have been identified, of which mostly interact with LC3 to induce engulfment of autophagosomes and subsequent internalization of lysosomes. However, under certain conditions lysosomes can act via micro-ER-phagy, a different program that allows lysosomes to independently internalise ER components^11,23^. In this study, scalp acupuncture treatment didn’t alter the expression of FAM134B. It is likely that FAM134B is not the ER-phagy receptor regulated by scalp acupuncture, thus we would continuously investigate whether other ER-phagy receptors, such as SEC62 and ATL3, involves in scalp acupuncture therapeutic effects. On the other hand, the results show that scalp acupuncture accelerated lysosomal endocytosis of ER components without augmenting LC3, a marker of macro-autophagy. Due to the interaction of ER-phagy receptors with LC3 plays an inevitable role in macro-ER-phagy, we surmise that scalp acupuncture treatment might not modulate ER stress via macro-ER-phagy. We also propose a hypothesis that scalp acupuncture inhibits ER stress by triggering micro-ER-phagy, which, however, need more evidences and further study to verify.

## Conclusion

In this study, we found that scalp acupuncture treatment administered based on vertex middle line and anterior oblique line of vertex temple needle insertion acupoint lines improved motor function of rats with MCAO-induced brain damage. Mechanistically, this therapeutic effect appeared to result from acupuncture-induced suppression of ER stress-triggered neuronal apoptosis. To our knowledge, this is the first study demonstrating involvement of ER-phagy in the therapeutic mechanism underlying scalp acupuncture therapeutic alleviation of cerebral ischemic stroke-induced neurological dysfunction. Nevertheless, additional investigations are needed to enhance our understanding of micro-ER-phagy pathway.

## Materials and methods

### Reagents

Reagents purchased from commercial sources included the following: pentobarbital sodium (Yipin Pharmaceutical Co., Ltd., Hebei, China); TTC staining reagent, 4% paraformaldehyde, diaminobenzidine, and hematoxylin (Solarbio Science & Technology Co., Beijing, China); fluorescein isothiocyanate (FITC)-conjugated secondary antibody and tetramethylrhodamine (TRITC)-conjugated secondary antibody (Invitrogen, CA, USA); tunicamycin (TM) (MCE, Shanghai, China); goat serum (Thermo Scientific, Rockford, IL, USA); primary antibodies specific for p-PERK, p-IRE1, ATF6, and p-JNK (Affinity Biosciences, Jiangsu, China); primary antibodies specific for LC3, CHOP, cleaved caspase-3, and cleaved caspase-9 (Proteintech, Wuhan, China); primary antibody specific for the KDEL peptide (Santa Cruz Biotechnology, Shanghai, China); primary antibody specific for FAM134B (Cell Signaling Technology, Danvers, MA, USA); and secondary antibodies (Bioss Antibodies, Beijing, China).

### Animals

Male Sprague–Dawley (SD) rats (8-weeks-old, weights 300 ± 20 g) were purchased from Yisi Experimental Animal Technology Co. (Changchun, China). Rats were housed in a dedicated specific pathogen-free (SPF) laboratory under 12-h light/12-h dark cycle conditions with free access to food and water. All animal experiments described in this study were conducted in accordance with ARRIVE guidelines and were approved by the Institutional Animal Care and Use Committee of Changchun University of Chinese Medicine (Changchun, China IACUC 2021092, May 8, 2021) prior to initiation of experiments.

### Experiments

Forty rats were randomly assigned to four groups (10 rats/group): control (CON) group, MCAO group, MCAO + acupuncture treatment (ACU) group, and MCAO + TM + acupuncture treatment (TM + ACU) group. Three weeks prior to MCAO surgery, TM (an ER stress agonist) was stereotactically injected into brains of TM + ACU group rats. Three weeks later, TM-injected rats received MCAO surgery with or without acupuncture treatment. The experiments schematically presented in Fig. 5A.

### MCAO surgery

First, rats were anaesthetised with 2% pentobarbital sodium (30 mg/kg) administered via intraperitoneal injection. Next, the common carotid artery (CCA), internal carotid artery (ICA), and external carotid artery (ECA) on the right side of each rat were carefully exposed. Thereafter, the CCA and ECA were ligated then a tiny opening was made in the ICA at a distance of 2 mm from the common cervical bifurcation. Next, a nylon embolus was inserted into the ICA using an 18–22-mm-long catheter (Shenzhen Reward Life Technology Co., China) to occlude the right middle cerebral artery (MCA). Rats in the CON group were anaesthetised then CCAs of CON group rats were exposed using the above mentioned procedures without MCAO induction.

### Scalp acupuncture treatment

After MCAO surgery, 0.3-mm-diameter acupuncture needles were inserted into left scalps of rats along the vertex middle line and the anterior oblique line of the vertex temple. Acupuncture needles remained in the scalp while they were twirled at 1 revolution per second for 1 min followed by a 4-min intermission, then these paired steps were repeated a total of 6 times during the 30-min operation. Acupuncture treatment was carried out once each day for 14 days.

### Neurological function test

Modified Zea-Longa score determinations, beam-walking tests, and screen-grabbing tests were conducted to assess neurological status and motor function of rats, as described in Tables 1, 2, and 3.

**Table 1:** Zea-Longa score.

Score	Method
0	No obvious neurological deficit
1	Forepaw not fully extended
2	Body of the rat circled to one side
3	Circling around and falling to one side
4	Unable to walk spontaneously and loss of consciousness

**Table 2:** Beam-walking test scale.

Score	Method
0	Unable to stay on the beam
1	Unable to go pass the beam
2	Go pass the beam but foot slip off more than 50%
3	Go pass the beam but foot slip off less than 50%
4	Pass the beam with no foot slip

**Table 3:** Screen-grabbing test scale.

Score	Method
0	Cannot grab the screen
1	Grab the screen for less than 10 s
2	Grab the screen for 10–19 s
3	Grab the screen for 20–29 s
4	Grab the screen for longer than 30 s

### TTC staining assay

After rats were sacrificed, brains were surgically removed as soon as possible and quickly frozen at − 80 °C for 5 min. Next, each brain was sectioned to generate 2-mm-thick coronal sections. Thereafter, the sections were soaked in 2% TTC staining reagent at 37 °C for 10 min in the dark then sections were flipped over and incubated under the same conditions for an additional 10 min. Next, brain sections generated from the same brain were placed in a row and imaged together. Thereafter, the proportion of brain area that was infarcted within each brain specimen was calculated based on the total area of infarcted brain tissue (white-coloured brain areas) divided by the total area of normal brain tissue (red-coloured brain areas).

### Brain edema measurement

The weight of each brain (total weight) was measured immediately after it was removed from the skull then all brains were frozen at − 80 °C overnight, lyophilised, and weighed again to obtain dry weights of freeze-dried brains. The difference between brain weight before and after lyophilisation reflected the extent of water retention (edema), as calculated using the following formula: (total weight-dry weight)/total weight × 100%.

### Preparation of paraffin sections

After surgical removal, brains were fixed in 4% paraformaldehyde at room temperature for 24 h. Next, brains were dehydrated by immersion in ethanol, vitrified by immersion in xylene, then vitrified brains were paraffin-embedded by immersing them in liquid paraffin. Thereafter, paraffinised brains were coronally sliced into 5-μm-thick sections at 2 mm after bregma, then the sections were dewaxed and stained with hematoxylin and eosin (H&E) stain, Nissl stain, or TUNEL stain. Results of H&E staining and Nissl staining were obtained by examining stained brain sections under an optical microscope (M8 Microscope, PreciPoint GmbH, Germany) and results of TUNEL staining were obtained by examining stained brain sections under a fluorescence microscope (LIONHEART, BioTek, VT, USA).

### Proteomics analysis

Brains were frozen in liquid nitrogen immediately after surgical removal then proteins in 50 mg of each rat’s right cortex were extracted using a lysis solution and digested with trypsin. Next, liquid chromatography–tandem mass spectrometry was conducted then proteins in brain tissues of MCAO and ACU groups were identified and compared using database searches (Maxquant v1.6.15.0) and bioinformatics analysis.

### Ultrastructural observations

Cortex on ischemic side was fixed in 2.5% glutaraldehyde (pH 7.4) for 2–4 h at 4 °C then samples were rinsed with 0.1 M phosphate buffer solution (PBS, pH 7.4) followed by post-fixation in 1% OsO_4_ (prepared in 0.1 M PBS) for 2 h at room temperature. Next, postfixed brains were subjected to graded dehydration, permeation, and embedding (acetone: embedding medium = 1:1) then the specimens were sliced into ultrathin (60–80-nm-thick) sections. Thereafter, brain sections were successively stained with 2% uranyl acetate for 15 min followed by staining with lead citrate for 15 min. Next, stained sections were analysed using a transmission electron microscope (TEM) System (Hitachi, Japan).

### Stereotactic injection of brain cortex

After rats were anaesthetised with 2% pentobarbital sodium (30 mg/kg) administered via intraperitoneal injection, rat scalp fur was sterilised then an incision was made in each scalp to gently expose the bregma. Next, each rat was immobilised using a Quintessential Stereotaxic Injector (Stoelting Co., IL, USA) with the bregma set to 0 (ML: 0.0, AP: 0.0, DV: 0.0) then 2 μL of 25 μM TM was injected into the brain cortex (ML: 2.0, AP: 1.2, DV: 1.5) at a rate of 0.5 μL/min. Meanwhile, rats of other groups were stereotactically injected with the same volume of DMSO. MCAO surgery and scalp acupuncture treatment were performed 3 weeks after stereotactic injection.

### Immunofluorescence and immuno-histochemical staining assays

Paraffinised brain sections were permeabilised by immersion in 0.3% Triton X-100 for 30 min at room temperature then sections were incubated in pH 10.0 Tris–HCl for 10 min at 100 °C. Next, sections were blocked by immersion in 5% goat serum for 1 h at 37 °C then were immersed in appropriate primary antibody solutions at 4 °C overnight. Thereafter, sections were incubated in appropriate secondary antibody solutions for 2 h at room temperature in the dark. Next, sections were photographed under a confocal microscope (Nikon C2 confocal, Japan) or were immersed in diaminobenzidine (DAB) for 10 min at room temperature, washed with PBS, soaked in hematoxylin for 0.5 min, washed with water, then dehydrated and photographed under an optical microscope.

### Statistical analysis

All triplicate data were analysed via ANOVA tests using IBM SPSS Statistics 23 software (IBM, NY, USA) and expressed as the mean ± standard deviation (GraphPad Prism 5, GraphPad Software, IL, USA). A value of p < 0.05 was considered statistically significant.

### Ethics declarations

All animal experiments described in this study were conducted according to established international ethical guidelines and were approved by the Institutional Animal Care and Use Committee of Changchun University of Chinese Medicine (Changchun, China IACUC 2021092, May 8, 2021) prior to initiation of experiments.

## Data Availability

All data of this study are available from the first author on reasonable request.

## Abbreviations

ER: Endoplasmic reticulum
UPR: Unfolded protein response
IRE1: Inositol-requiring protein-1
PERK: Protein kinase R-like endoplasmic reticulum kinase
ATF6: Activating transcription factor-6
JNK: C-Jun NH 2-terminal kinase
CHOP: C/EBP homologous protein
LC3B: Microtubule associated protein light chain 3B
FAM134B: Family with sequence similarity 134, member B
RTN3L: Reticulon 3 long
CCPG1: Cell-cycle progression gene 1
SEC62: SEC62 homologue
TEX264: Testis-expressed 264
ATL3: Atlastin 3
MCAO: Middle cerebral artery occlusion
TTC: 2,3,5-Triphenyltetrazolium chloride
CCA: Common carotid artery
ICA: Internal carotid artery
ECA: External carotid artery
TM: Tunicamycin
ACU: Acupuncture
PBS: Phosphate buffer solution
H&E: Hematoxylin–eosin
TUNEL: Terminal deoxynucleotidyl transferase biotin-dUTP nick end labeling
ERAD: ER-associated protein degradation
